# Supplementary material for: Your best day: An interactive app to translate how time reallocations within a 24-hour day are associated with health measures
Source: PLoS One. 2022 Sep 7;17(9):e0272343. doi: 10.1371/journal.pone.0272343 (PMC9451088; doi:10.1371/journal.pone.0272343)
Supplement: S1 Fig — (PDF) [file pone.0272343.s001.pdf]

Supplementary File 2: Shiny app detail and schematic diagram

The Shiny app developed in the current study has three components (i.e. R scripts) that communicate with each other (Fig S2): (1) the user interface (ui.R), (2) the server (server.R) and (3) the global environment (global.R). Below are annotated R scripts used for the Shiny app – these can also be downloaded from GitHub https://github.com/dotdum/realloc_adventure.

The user interface (ui.R) describes the appearance of the app. For our app, this script defines the number of pages (tabs), how the pages are laid out, what text the user will see, and how the user will enter their information. Any user input needed to update the app's output is called a reactive object. For example, if the user wished to exchange 30 minutes of physical activity for screen time, this information was stored as a reactive object in the ui.R script. The information provided by the user is then made available to the server for subsequent computation.

The server.R script contains instructions for how user input is processed and what output to return to the user interface. For example, if the user would wish to exchange 30 minutes of physical activity for screen time, the server would use this reallocation to compute the estimated difference in outcomes before returning output for the user to see.

Both the server.R and ui.R scripts call on the global.R script, which acts like a "bookshelf" for any warning messages. For example, a warning was set to appear if the user's daily time-use profile did not add to a total of 1440 minutes or 24 hours. The global script also stores the pre-defined R functions needed to run the application. One of these functions replaced any user-specified zero amounts of time with 3.25 minutes (65% of the 5-minute sampling frame [33]) to allow the calculation of isometric log-ratio coordinates. Alternatively, this function could be altered to not allow the user to input any zero values.

The compositional regression coefficients from the three models defined below (i.e. with body fat percentage, psychosocial health, and academic performance as outcome variables) were used to compute the estimated differences in outcomes for user-defined time-reallocations.

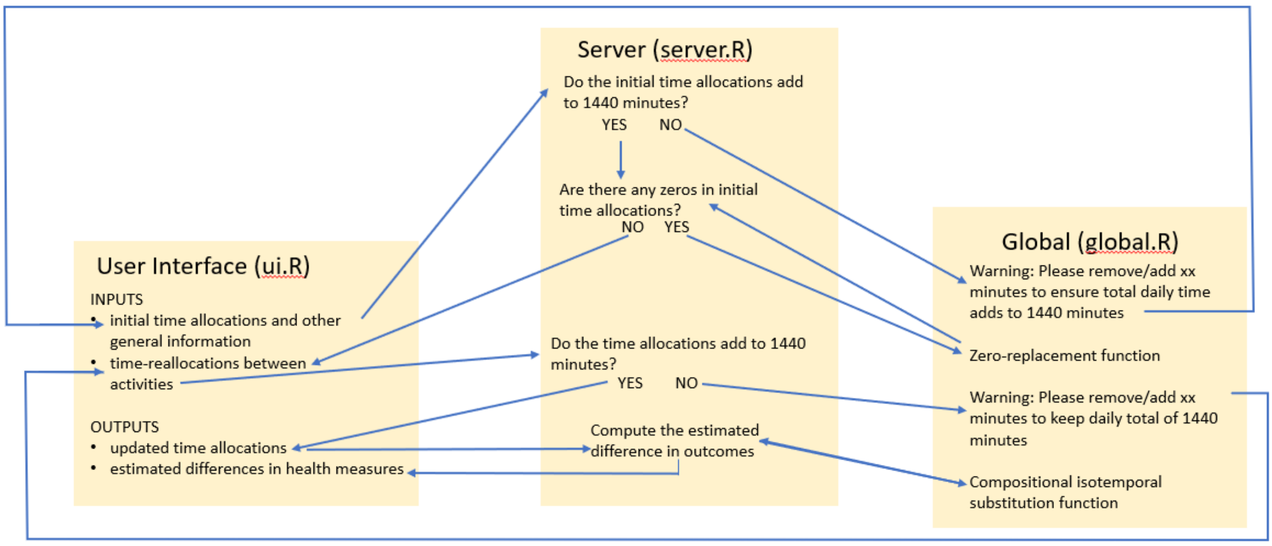

Figure S2. Simplified schematic representation of the Shiny app
