# Supplementary material for: Your best day: An interactive app to translate how time reallocations within a 24-hour day are associated with health measures
Source: PLoS One. 2022 Sep 7;17(9):e0272343. doi: 10.1371/journal.pone.0272343 (PMC9451088; doi:10.1371/journal.pone.0272343)
Supplement: S1 Table — (PDF) [file pone.0272343.s002.pdf]

Supplementary File 4

Compositional model summaries (ANOVA Table: Type III tests)

		**%body fat**[a] *n*=1672		**PedsQL psychosocial** *n*=1679		**NAPLAN writing performance** *n*=1294	
**Variable**	**Df**	**F**	***P***	**F**	***p***	**F**	***p***
Intercept	1	175.0	<0.001	23.9	<0.001	81.0	<0.001
Sex	1	40.9	<0.001	8.7	0.003	30.0	<0.01
Age	1	23.3	<0.001	0.4	0.519	2.9	0.087
SEP	1	23.6	<0.001	15.3	<0.001	69.5	<0.001
Pubertal stage	4	5.9	<0.001	3.2	0.012	1.2	0.319
Ilrs[b]	27	2.7	<0.001	4.0	<0.001	2.8	<0.001

[a]log transformed; [b]includes second-order polynomial term; *ilr* = isometric log ratio of time-use composition; SEP = socioeconomic position
