# Supplementary material for: Your best day: An interactive app to translate how time reallocations within a 24-hour day are associated with health measures
Source: PLoS One. 2022 Sep 7;17(9):e0272343. doi: 10.1371/journal.pone.0272343 (PMC9451088; doi:10.1371/journal.pone.0272343)
Supplement: S1 Data — (PDF) [file pone.0272343.s003.pdf]

Supplementary File 3: Shiny App code: `realloc_adventure`

18 Aug 2021

Table of Contents

global.R script 1
ui.R script 4
server.R script 9

global.R script

```
# ++++ libs ++++

# Load package libraries
library(shiny)
require(shinydashboard)
library(shinyjs)
library(dplyr)
library(ggplot2)
library(plotly)
library(compositions)
library(foreach)


# ++++ debug boolean ++++

####################################
### set this TRUE for debug mode ###
####################################

debug_mode <- TRUE


# ++++ load rdata ++++

# Source pre-created model elements
rdat_fls <- c("dat/lm_ln_fat.RData", "dat/lm_psy.RData", "dat/lm_aca.RData")

fl_exists <- file.exists(rdat_fls)
if (!all(fl_exists)) {
  stop(
    paste0(
      "Please make sure the file(s) ",
      paste(rdat_fls[!fl_exists], collapse = ", "),
      " are available to the app. Currently not found."
    )
  )
}

# from "dat/lm_ln_bf.RData", load the objects: beta_ln_bf, vcov_ln_bf, resdf_ln_bf
# from "dat/lm_psy.RData", load the objects:     beta_psy,   vcov_psy,   resdf_psy
# from "dat/lm_aca.RData", load the objects:     beta_aca,   vcov_aca,   resdf_aca
for (f in rdat_fls) {
  load(f)
}


# Define activity names and number of activities
```

```r
activity_nms <- c('Sleep', 'Screen', 'PA', 'QuietT', 'PassiveTrans', 'School', 'Domestic_SelfCare')
nact <- length(activity_nms)

# ++++ define error messages ++++

# Negative composition error
err1.string <- paste0(
  "Time allocation does not add up to 24 hours."
)

err2.string <- paste0(
  "Reallocated times must add up to zero hours."
)


# ++++ server_side_fns ++++

sanitise_ilrs <- function(x) {

  if ("rmult" %in% class(x)) {
    class(x) <- NULL # remove "rcomp" class, will either result in numeric vector or matrix
    attr(x, "orig") <- NULL # remove original composition info (issue with indexes)
    if ("numeric" %in% class(x)) { # if vector turn into 1 row matrix
      x <- matrix(x, nrow = 1, dimnames = list(NULL, names(x)))
    }
  }
  return(x)

}


poly2 <- function(x) {

  # make sure is matrix
  x <- sanitise_ilrs(x)

  n <- ncol(x)
  cnames <- colnames(x)

  if (is.null(cnames)) {
    cnames <- paste0("c", 1L:n)
  }

  # get all tuples of (j,k) where j <= k
  tups <- subset(expand.grid(j = 1:n, k = 1:n), j <= k)
  tups <- tups[order(tups$j, tups$k), ] # make sure consistent ordering
  j <- tups$j
  k <- tups$k

  # drop = FALSE is to make sure 1 row matrices don't become vectors
  sq_out <- x[, j, drop = FALSE] * x[, k, drop = FALSE]
  colnames(sq_out) <- paste0(cnames[j], ":", cnames[k])

  return(cbind(x, sq_out))

}



make_x0 <- function(betas, ilrs, sex, age, sep, pub) {

  beta_nms <- names(betas)
  ilr_nms <- beta_nms[grepl("^ilrs_", beta_nms)]

  ilrs <- sanitise_ilrs(ilrs)

  # beta_nms <- gsub("ilrs_", "", beta_nms, fixed = TRUE)
  # ilr_nms <- gsub("ilrs_", "", ilr_nms, fixed = TRUE)
  p <- length(betas)
```

```r
  # initialise model matrix
  x0 <- matrix(0, nrow = nrow(ilrs), ncol = p, dimnames = list(NULL, beta_nms))

  # populate model matrix intercept column
  x0[, "(Intercept)"] <- 1

  # populate ilr columns in model matrix
  x0_ilrs <- poly2(ilrs)
  x0_ilrs_nms <- {colnames(x0_ilrs) <- paste0("ilrs_", colnames(x0_ilrs))}
  ### testing
  # print(x0_ilrs_nms)
  # print(ilr_nms)

  if (!(all(x0_ilrs_nms %in% ilr_nms) & all(ilr_nms %in% x0_ilrs_nms))) {
    stop("The ilrs in the model and the supplied ilrs have differing names (or possibly differ in
number)")
  }
  # if in different orders, make x0_ilrs order the same as ilr_nms
  if (!all(x0_ilrs_nms == ilr_nms)) {
    reorder <- match(ilr_nms, x0_ilrs_nms)
    x0_ilrs <- x0_ilrs[reorder]
    x0_ilrs_nms <- colnames(x0_ilrs)
    if (!all(x0_ilrs_nms == ilr_nms)) {
      stop("ilr names supplied in `ilrs` could not be reordered to match the ilr names in `mod`")
    }
  }

  x0[, ilr_nms] <- x0_ilrs

  # populate covariate columns in model matrix
  # covariates in model matrix are dealt with in a vectorised way for when there is more than one row in
ilrs
  x0[, "cov.sex2"] <- as.numeric(as.character(sex) == "2")
  x0[, "cov.age"] <- age
  x0[, "cov.sep"] <- sep
  puberty_vals <-
    as.integer(
      gsub(
        "cov.puberty",
        "",
        beta_nms[grepl("cov.puberty", beta_nms, fixed = TRUE)],
        fixed = TRUE
      )
    )
  pub <- as.character(pub)
  for (i in puberty_vals) {
    x0[, paste0("cov.puberty", i)] <- as.numeric(pub == as.character(i))
  }

  return(x0)

}


get_pred_bounds <- function(betas, x0, beta_vcov, df, bound = 0, alpha = 0.05) {
  betas <- matrix(betas, ncol = 1)
  crit_val <- qt(1 - alpha / 2, df = df)
  foreach(i = 1:nrow(x0), .combine = c) %do% {
    x0_i <- x0[i, , drop = FALSE]
    as.numeric(x0_i %*% betas) +
      bound * crit_val * sqrt(as.numeric(x0_i %*% beta_vcov %*% t(x0_i)))
  }
}




# For confidence intervals of the difference in lognormal predictions see:
```

```r
# Guang Yong Zou, Julia Taleban, Cindy Y. Huo (2009)
# "Confidence interval estimation for lognormal data with application to health economics"
# This implements equation (4) of Zou et al.
diff_lognorm_cis <- function(m1, l1, u1, m2, l2, u2, r = 0) {

  ctr <- m1 - m2

  marg_lo <-
    sqrt(
      (m1 - l1) ^ 2 + (u2 - m2) ^ 2 - 2 * r * (m1 - l1) * (u2 - m2)
    )
  marg_hi <-
    sqrt(
      (u1 - m1) ^ 2 + (m2 - l2) ^ 2 - 2 * r * (u1 - m1) * (m2 - l2)
    )

  ci <- c(ctr - marg_lo, ctr + marg_hi)

  return(ci)

}



shiny_gg_theme <- function() {
  theme(
    legend.position = "none",
    axis.title = element_text(size = 12),
    axis.text = element_text(size = 12, colour = "grey50"),
    strip.text = element_text(size = 12),
    plot.title = element_text(size = 14),
    plot.subtitle = element_text(size = 10, colour = "grey50")
  )
}
```

ui.R script

```r
# ++++ Header ++++

header <- dashboardHeader(
    titleWidth = 750,
  title = "Compositional Isotemporal Substitution: Time-Reallocation Interface"
)  #dashboardHeader

# ++++ Sidebar ++++

# Determines how many tabs exist in the body of the ui

sidebar <- dashboardSidebar(
  width = 175, #Width of sidebar the same as width of header
  useShinyjs(),
  # Sidebar options
  sidebarMenu(
    menuItem(
      "Initial time-use",
      tabName = "participant-tab",
      icon = icon("child")
    ),  # menuItem.participant-tab
    menuItem(
      "Specify reallocations",
      tabName = "time-tab",
      icon = icon("time", lib = "glyphicon")
    )  # menuItem.time-tab
  )  # sidebarMenu
)  # dashboardSidebar


# ++++ Body ++++
```

```r
# Main content of each tab, as determined by sidebar

body <- dashboardBody(
  tabItems(

    ### participant Information Tab

    tabItem(
      tabName = "participant-tab",

      # Covariate and Initial Composition Input
      fluidRow(

        # general input column (left)

        box(
          title = "General Information", width = 3, status = "primary",
          column(
            width = 12,
            radioButtons(
              "sex", "Sex",
              choices = list("Male" = 1, "Female" = 2), inline = FALSE
            )  # radioButtons.sex
          ), # column.sex
          column(
            width = 12,
            numericInput(
              "age", "Age (years)",
              value = 12
            )  # numericInput.age
          ) # column.age

        ),  # box.participant-input

      # time allocations input column (centre)

        box(
          title = "Please Provide Current Time Allocations", width = 6, status = "primary",

          fluidRow(
            column(
              width = 6,
              strong("Sleep"),
              numericInput(
                "initSleep", "(hours)", width = "100%",
                value = 11, step = 1, min = 0, max = 23
              ),  # numericInput.initSleep.hours
            ),
            column(
              width = 6,
              br(),
              numericInput(
                "initSleepmin", "(mins)", width = "100%",
                value = 50, step = 1, min = 0, max = 59
              )  # numericInput.initSleep.minutes
            )
          ),  # fluidRow.initSleep

          fluidRow(
            column(
              width = 6,
              strong("Screen"),
              numericInput(
                "initScreen", "(hours)", width = "100%",
                value = 2, step = 1, min = 0, max = 23
              )  # numericInput.initScreen
            ),
            column(
```

```
      width = 6,
      br(),
      numericInput(
        "initScreenmin", "(mins)", width = "100%",
        value =55 , step = 1, min = 0, max = 59
      )  # numericInput.initScreen.minutes
    )
  ),  # fluidRow.initScreen

  fluidRow(
    column(
      width = 6,
      strong("Physical Activity"),
      numericInput(
        "initPA", "(hours)", width = "100%",
        value = 1, step = 1, min = 0, max = 23
      ) # numericInput.initPA.hours
    ),
    column(
      width = 6,
      br(),
      numericInput(
        "initPAmin", "(mins)", width = "100%",
        value = 57, step = 1, min = 0, max = 59
      )  # numericInput.initPA.minutes
    )
  ),  # fluidRow.initPA

  fluidRow(
    column(
      width = 6,
      strong("Quiet Time"),
      numericInput(
        "initQuietT", "(hours)", width = "100%",
        value = 1, step = 1, min = 0, max = 23
      ) # numericInput.initQuietT
    ),
    column(
      width = 6,
      br(),
      numericInput(
        "initQuietTmin", "(mins)", width = "100%",
        value = 11, step = 1, min = 0, max = 59
      )  # numericInput.initQuietT.minutes
    )
  ),  # fluidRow.initQuietT

  fluidRow(
    column(
      width = 6,
      strong("Passive Transport"),
      numericInput(
        "initPassiveTrans", "(hours)", width = "100%",
        value = 0, step = 1, min = 0, max = 23
      )  # numericInput.initPassiveTrans
    ),
    column(
      width = 6,
      br(),
      numericInput(
        "initPassiveTransmin", "(mins)", width = "100%",
        value = 35, step = 1, min = 0, max = 59
      )  # numericInput.initPassiveTrans.minutes
    )
  ),  # fluidRow.initSchool

  fluidRow(
    column(
      width = 6,
```

```
          strong("School-Related"),
          numericInput(
            "initSchool", "(hours)", width = "100%",
            value = 2, step = 1, min = 0, max = 23
          )  # numericInput.initSchool
        ),
        column(
          width = 6,
          br(),
          numericInput(
            "initSchoolmin", "(mins)", width = "100%",
            value = 9, step = 1, min = 0, max = 59
          )  # numericInput.initSchool.minutes
        )
      ),  # fluidRow.initSchool

      fluidRow(
        column(
          width = 6,
          strong("Domestic & Self Care"),
          numericInput(
            "initDomestic_SelfCare", "(hours)", width = "100%",
            value = 3, step = 1, min = 0, max = 23
          )  # numericInput.initScreen
        ),
        column(
          width = 6,
          br(),
          numericInput(
            "initDomestic_SelfCaremin", "(mins)", width = "100%",
            value = 23, step = 1, min = 0, max = 59
          )  # numericInput.initDomestic_SelfCare.minutes
        ),
      ), # fluidRow.initDomestic_SelfCare

      # error msg if not == 24 hours
      div(textOutput("err1"), style = "color: red")
    ), # box.time-input

    # advanced input column (right)

    box(
      title = "Advanced Information", width = 3, status = "primary",

      column(
        width = 12,
        radioButtons(
          "puberty", "Pubertal Stage",
          choices = list("Pre-pubertal" = 1, "Early Puberty" = 2,
          "Mid-pubertal" = 3, "Late Puberty" = 4, "Post-pubertal" = 5),
          selected=3, inline=FALSE
        )  # radioButtons.puberty
      ),  # column.puberty column(width = 6,

      column(
        width = 12,
        numericInput(
          "sep", "SES (z-score)",
          value = 0, min = -3, max = 3, step = 0.5
        )  # numericInput.sep
      )  # column.sep
    ) # box advanced input
  ) # fluidRow
), # participant-tab

### Time Re-Allocation Tab
  tabItem(
    tabName = "time-tab",
```

```
      # Time re-allocation sliders

      column(width = 6,
        box(
          title = "Provide Time-Reallocations", width = 12,
          sliderInput(
            "Sleep", "Sleep",
            value = 0, min = -60, max = 60, ticks = FALSE
          ), # sliderInput.Sleep
          sliderInput(
            "Screen", "Screen Time",
            value = 0, min = -60, max = 60, ticks = FALSE
          ), # sliderInput.DomSoc
          sliderInput(
            "PA", "Physical Activity",
            value = 0, min = -60, max = 60, ticks = FALSE
          ), # sliderInput.PA
          sliderInput(
            "QuietT", "Quiet Time",
            value = 0, min = -60, max = 60, ticks = FALSE
          ), # sliderQuietT
          sliderInput(
            "PassiveTrans", "Passive Transport",
            value = 0, min = -60, max = 60, ticks = FALSE
          ), # sliderInput.PassiveTrans
          sliderInput(
            "School", "School-Related",
            value = 0, min = -60, max = 60, ticks = FALSE
          ), # sliderInput.School
          sliderInput(
            "Domestic_SelfCare", "Domestic & SelfCare",
            value = 0, min = -60, max = 60, ticks = FALSE
          ),  # sliderInput.Domestic_SelfCare
          div(textOutput("err2"), style = "color: red"),
          div(textOutput("err3"), style = "color: orange"),
          actionButton("reset_sliders", label = "Reset sliders")
        ),# box.changetime,

        box(
          width = 12, align = "center",
          title = "New daily time composition",
          plotlyOutput("time_use_plot_1"),
        ) # box.treemap of time-compositions

      ), # column.left

      # OUTCOMES

      column(
        width = 6, align = "center",

        box(
          id = "colour_out",
          width = 12,
          uiOutput("ui1"),
          uiOutput("ui2"),
          uiOutput("ui3")
        ),

        box(
          id = "showhide",
          width = 12,
          actionButton(inputId = "sh_but", label = "show / hide advanced output"),
        ),

        box(id = "plot1",
          width = 12,
          plotOutput("pred_plot_1"),
          plotOutput("pred_plot_2")
```

```
        )
      ) # column.right
    )   # time-tab
  )  # tabItems
)  # dashboardBody

#  ++++ UI_end ++++

dashboardPage(header, sidebar, body)
```

server.R script

```
# set where errors go
options(shiny.error = browser)

# all reactive values, output and ui elements
server <- function(input, output, session) {

  # shinyjs call to hide output until requested
  shinyjs::hide(id = "plot1")

  # ++++ composition_funcs ++++


  # Initial Composition and Outcome Values
  # Reactive objects based on user data from the participant-tab
  # Define initial times (hours plus minutes)

  init.time <- reactive({

    init_df <-
        data.frame(
        Sleep = input$initSleep + input$initSleepmin/60,
        Screen = input$initScreen + input$initScreenmin/60,
        PA = input$initPA + input$initPAmin/60,
        QuietT = input$initQuietT + input$initQuietTmin/60,
        PassiveTrans = input$initPassiveTrans + input$initPassiveTransmin/60,
        School = input$initSchool + input$initSchoolmin/60,
        Domestic_SelfCare = input$initDomestic_SelfCare + input$initDomestic_SelfCaremin/60
      )

    # check no NA values present: turn into 0s if so
    if (any(is.na(init_df))) {

      if (debug_mode) {
        print(init_df)
      }

      init_df[, c(is.na(init_df))] <- 0

      if (debug_mode) {
        print(init_df)
      }

    }

    # check for 0 values as we need to add 65% of min to those values for ilrs to work
    lt0_df <- unlist(init_df) <= 0

    sum_chk <- rowSums(init_df)

    if (any(lt0_df)) {

    # smallest non-zero time is 65% of a minute
      quantum_of_time <- 0.65 / 60

      n_lt0 <- sum(lt0_df)
      n_gt0 <- sum(!lt0_df)
```

```
    # 65% of a minute, smallest allocation, give to the poor
    init_df[, lt0_df] <-  quantum_of_time
    # take from the rich
    init_df[, !lt0_df] <- init_df[, !lt0_df] - n_lt0 * quantum_of_time / n_gt0

    # make sure still sums to 1 day after small tweaks to account for 0s
    if (sum_chk != rowSums(init_df)) {
      stop("Re-allocaiton of small time units to negate 0s unsuccessful")
    }

  }

  if (debug_mode) {
    print(init_df)
  }

  return(init_df)

})

# Define initial composition
# Based on input if it adds up to 24, otherwise based on mean composition

##it would be better if the user was prompted to make this sum to 24 h##**
init.comp <- reactive({
  if (sum(init.time()) == 24) {
    acomp(init.time())
  } else {
    m.comp
  }
})

# Capture reallocated times
reall.time <- reactive({
  c(
    input$Sleep,
    input$Screen,
    input$PA,
    input$QuietT,
    input$PassiveTrans,
    input$School,
    input$Domestic_SelfCare
  )
})


# Define reactive times
Rtime <- reactive({
  if (sum(reall.time()) == 0) {
    out <- init.time()[1,] + reall.time()/60
    names(out) <- activity_nms
    return(out)
  } else {
    init.time()
  }
})

# Define reactive composition
Rcomp <- reactive({
  acomp(Rtime())
})

# Define error text that checks whether initial time inputs adds up to 24
output$err1 <- renderText({
  if (sum(init.time()) != 24) {
    diff <- 24 - sum(init.time())
    more.less <- ifelse(diff > 0, "add", "remove")
    hours <- floor(abs(diff))
    mins <- round((abs(diff) - hours)*60)
```

```
      hours.mins <- paste0(
      ifelse(hours == 0, "", paste(hours, "hour(s)")),
      ifelse(hours == 0 | mins == 0, "", " and "),
      ifelse(mins == 0, "", paste(mins, "minute(s)"))
    )
    paste(err1.string, "Please", more.less, hours.mins)
  } else {
    " "
  }
})

# Define error text that checks whether reallocation time inputs adds up to 0
output$err2 <- renderText({
  if (sum(reall.time()) != 0) {
    diff <- 0 - sum(reall.time())
    more.less <- ifelse(diff > 0, "add", "remove")
    mins <- abs(diff)
    paste(err2.string, "Please", more.less, mins, "minute(s)")
  } else {
    " "
  }
})

# Define error text that checks whether reallocation time inputs have compositions > 0
output$err3 <- renderText({
  rt_vec <- Rtime()
  comp_nms <- names(rt_vec)
  comp_vls_lt0 <- rt_vec <= 0

  if (any(comp_vls_lt0)) {
    paste0(
      "Currently there are time-use categories with time(s) not greater than 0 mins. \n",
      "This is because the re-allocation of time exceeds the corresponding ",
      "starting time in its time-use category. \n",
      "The listed time-use categories below provide the mintutes required ",
      "for there to be positive time in categories (minutes in brackets): \n",
      paste(
        paste0(
          comp_nms[comp_vls_lt0],
          " (",
          round(-rt_vec[comp_vls_lt0] * 60 + 1, 0),
          " minutes)"
        ),
        collapse = ",\n"
      )
    )
  } else {
    " "
  }
})


observeEvent(input$reset_sliders, {

  updateSliderInput(session, 'Sleep' ,value = 0)
  updateSliderInput(session, 'Screen' ,value = 0)
  updateSliderInput(session, 'PA' ,value = 0)
  updateSliderInput(session, 'QuietT' ,value = 0)
  updateSliderInput(session, 'PassiveTrans' ,value = 0)
  updateSliderInput(session, 'School' ,value = 0)
  updateSliderInput(session, 'Domestic_SelfCare' ,value = 0)

})

# ++++ composition_output ++++

output$time_use_plot_1 <- renderPlotly({

  hrs_comp <- as.numeric(Rcomp()) * 24
```

```
  mins_comp <- hrs_comp * 60

  prnts <- rep("", length(activity_nms))
  txt_lbs <- sprintf("%2.1f hrs\n(%2.0f mins)", hrs_comp, mins_comp)

  if (debug_mode) {
    # print(Rcomp())
    # print(as.numeric(Rcomp()))
    print(hrs_comp); print(mins_comp); print(prnts); print(txt_lbs)
  }

  # https://plotly.com/r/treemaps/
  plot_ly(
    type = "treemap",
    labels = activity_nms,
    parents = prnts,
    values = mins_comp,
    text = txt_lbs
  )

})


# --- Model_output ++++

# Predicted outcomes from selected models on result-tab
# Define predictions for initial composition


# ++++ fat_outc ++++


init_pred_fat <- reactive({

  init_ilrs <- ilr(init.comp())
  names(init_ilrs) <- paste0("ilr", 1:length(init_ilrs))

  x0_init <- make_x0(beta_ln_fat, init_ilrs, input$sex, input$age, input$sep, input$puberty)

  out_pr <- get_pred_bounds(beta_ln_fat, x0_init, vcov_ln_fat, resdf_ln_fat, bound =  0, alpha = 0.05)
  out_lo <- get_pred_bounds(beta_ln_fat, x0_init, vcov_ln_fat, resdf_ln_fat, bound = -1, alpha = 0.05)
  out_hi <- get_pred_bounds(beta_ln_fat, x0_init, vcov_ln_fat, resdf_ln_fat, bound = +1, alpha = 0.05)

  out_init <- c(out_pr, out_lo, out_hi)
  out_init <- exp(out_init) # because log transformed outcome

  if (debug_mode) {
    print(out_init)
  }

  return(out_init)

})


# Define predictions for reallocation composition
reall_pred_fat <- reactive({

  realloc_ilrs <- ilr(Rcomp())
  names(realloc_ilrs) <- paste0("ilr", 1:length(realloc_ilrs))

  x0_realloc <- make_x0(beta_ln_fat, realloc_ilrs, input$sex, input$age, input$sep, input$puberty)

  out_pr <- get_pred_bounds(beta_ln_fat, x0_realloc, vcov_ln_fat, resdf_ln_fat, bound =  0, alpha =
0.05)
  out_lo <- get_pred_bounds(beta_ln_fat, x0_realloc, vcov_ln_fat, resdf_ln_fat, bound = -1, alpha =
0.05)
  out_hi <- get_pred_bounds(beta_ln_fat, x0_realloc, vcov_ln_fat, resdf_ln_fat, bound = +1, alpha =
0.05)
```

```r
  out_realloc <- c(out_pr, out_lo, out_hi)
  out_realloc <- exp(out_realloc) # because log transformed outcome

  if (debug_mode) {
    print(out_realloc)
  }

  return(out_realloc)

})


# Define predictions for reallocation composition
delta_pred_fat <- reactive({

  init_ilrs <- ilr(init.comp())
  names(init_ilrs) <- paste0("ilr", 1:length(init_ilrs))

  realloc_ilrs <- ilr(Rcomp())
  names(realloc_ilrs) <- paste0("ilr", 1:length(realloc_ilrs))

  if (debug_mode) {
    print(init_ilrs)
    print(realloc_ilrs)
    print(init_ilrs - realloc_ilrs)
  }

  # if the reallocation ilrs are the same as initial, then no prediction need be done
  if (sum(abs(init_ilrs - realloc_ilrs)) < 1e-6) {
    return(rep(0, 3))
  }

  # for confidence intervals of the difference in lognormal predictions see:
  # Guang Yong Zou, Julia Taleban, Cindy Y. Huo (2009)
  # "Confidence interval estimation for lognormal data with application to health economics"


  # let x_b be initial/before and x_a be after realloc

  # also note that if y1 and y2 have a bivariate normal distribution where
  # (y1, y2) ~ N([mu1, m2], [se^2 x1^T (X^T X)^{-1} x1, se^2 x2^T (X^T X)^{-1} x2, rho]
  # THEN rho = x1^T (X^T X)^{-1} x2 / sqrt(x1^T (X^T X)^{-1} x1) * sqrt(x2^T (X^T X)^{-1} x2)

  # pred vals exponentiated
  exp_y_a <- reall_pred_fat()
  exp_y_b <- init_pred_fat()

  # back to log scale and normally distributed
  y_a <- log(exp_y_a)
  y_b <- log(exp_y_b)

  # need these prediction coefficients to calculate correlation and variation
  x_a <- make_x0(beta_ln_fat, realloc_ilrs, input$sex, input$age, input$sep, input$puberty)
  x_b <- make_x0(beta_ln_fat, init_ilrs, input$sex, input$age, input$sep, input$puberty)

  sigma_a <- sqrt(as.numeric(x_a %*% vcov_ln_fat %*% t(x_a)))
  sigma_b <- sqrt(as.numeric(x_b %*% vcov_ln_fat %*% t(x_b)))

  # equations (8) and (9) of Zou et al.
  rho <-
    as.numeric(x_b %*% vcov_ln_fat %*% t(x_a)) / (sigma_a * sigma_b)

  r <-
    (exp(rho * sigma_b * sigma_a) - 1) /
      sqrt((exp(sigma_b ^ 2) - 1) * (exp(sigma_a ^ 2) - 1))

  if (debug_mode) {
    print(paste("rho:", rho))
```

```
    print(paste("r:", r))
  }

  # equation (4) of Zou et al.
  diff_ci <-
    diff_lognorm_cis(
      m1 = exp_y_a[1],
      l1 = exp_y_a[2],
      u1 = exp_y_a[3],
      m2 = exp_y_b[1],
      l2 = exp_y_b[2],
      u2 = exp_y_b[3],
      r = r
    )

  exp_out_delta <- c(exp_y_a[1] - exp_y_b[1], diff_ci[1], diff_ci[2])

  return(exp_out_delta)

})

perc_change_fat <- reactive({ 100 * delta_pred_fat()[1] / init_pred_fat()[1] })


# ++++ psy_outc ++++


init_pred_psy <- reactive({

  init_ilrs <- ilr(init.comp())
  names(init_ilrs) <- paste0("ilr", 1:length(init_ilrs))

  x0_init <- make_x0(beta_psy, init_ilrs, input$sex, input$age, input$sep, input$puberty)

  out_pr <- get_pred_bounds(beta_psy, x0_init, vcov_psy, resdf_psy, bound =  0, alpha = 0.05)
  out_lo <- get_pred_bounds(beta_psy, x0_init, vcov_psy, resdf_psy, bound = -1, alpha = 0.05)
  out_hi <- get_pred_bounds(beta_psy, x0_init, vcov_psy, resdf_psy, bound = +1, alpha = 0.05)

  out_init <- c(out_pr, out_lo, out_hi)

  if (debug_mode) {
    print(out_init)
  }

  return(out_init)

})


# Define predictions for reallocation composition
reall_pred_psy <- reactive({

  realloc_ilrs <- ilr(Rcomp())
  names(realloc_ilrs) <- paste0("ilr", 1:length(realloc_ilrs))

  x0_realloc <- make_x0(beta_psy, realloc_ilrs, input$sex, input$age, input$sep, input$puberty)

  out_pr <- get_pred_bounds(beta_psy, x0_realloc, vcov_psy, resdf_psy, bound =  0, alpha = 0.05)
  out_lo <- get_pred_bounds(beta_psy, x0_realloc, vcov_psy, resdf_psy, bound = -1, alpha = 0.05)
  out_hi <- get_pred_bounds(beta_psy, x0_realloc, vcov_psy, resdf_psy, bound = +1, alpha = 0.05)

  out_realloc <- c(out_pr, out_lo, out_hi)

  if (debug_mode) {
    print(out_realloc)
  }

  return(out_realloc)
```

```r
})


# Define predictions for reallocation composition
delta_pred_psy <- reactive({

  init_ilrs <- ilr(init.comp())
  names(init_ilrs) <- paste0("ilr", 1:length(init_ilrs))

  realloc_ilrs <- ilr(Rcomp())
  names(realloc_ilrs) <- paste0("ilr", 1:length(realloc_ilrs))

  x0_delta <-
  make_x0(beta_psy, realloc_ilrs, input$sex, input$age, input$sep, input$puberty) -
  make_x0(beta_psy, init_ilrs, input$sex, input$age, input$sep, input$puberty)

  out_pr <- get_pred_bounds(beta_psy, x0_delta, vcov_psy, resdf_psy, bound =  0, alpha = 0.05)
  out_lo <- get_pred_bounds(beta_psy, x0_delta, vcov_psy, resdf_psy, bound = -1, alpha = 0.05)
  out_hi <- get_pred_bounds(beta_psy, x0_delta, vcov_psy, resdf_psy, bound = +1, alpha = 0.05)

  out_delta <- c(out_pr, out_lo, out_hi)

  if (debug_mode) {
    print(x0_delta)
    print(out_delta)
  }

  return(out_delta)

})

perc_change_psy <- reactive({ 100 * delta_pred_psy()[1] / init_pred_psy()[1] })


# ++++ aca_outc ++++


init_pred_aca <- reactive({

  init_ilrs <- ilr(init.comp())
  names(init_ilrs) <- paste0("ilr", 1:length(init_ilrs))

  x0_init <- make_x0(beta_aca, init_ilrs, input$sex, input$age, input$sep, input$puberty)

  out_pr <- get_pred_bounds(beta_aca, x0_init, vcov_aca, resdf_aca, bound =  0, alpha = 0.05)
  out_lo <- get_pred_bounds(beta_aca, x0_init, vcov_aca, resdf_aca, bound = -1, alpha = 0.05)
  out_hi <- get_pred_bounds(beta_aca, x0_init, vcov_aca, resdf_aca, bound = +1, alpha = 0.05)

  out_init <- c(out_pr, out_lo, out_hi)

  if (debug_mode) {
    print(out_init)
  }

  return(out_init)

})


# Define predictions for reallocation composition
reall_pred_aca <- reactive({

  realloc_ilrs <- ilr(Rcomp())
  names(realloc_ilrs) <- paste0("ilr", 1:length(realloc_ilrs))

  x0_realloc <- make_x0(beta_aca, realloc_ilrs, input$sex, input$age, input$sep, input$puberty)

  out_pr <- get_pred_bounds(beta_aca, x0_realloc, vcov_aca, resdf_aca, bound =  0, alpha = 0.05)
  out_lo <- get_pred_bounds(beta_aca, x0_realloc, vcov_aca, resdf_aca, bound = -1, alpha = 0.05)
```

```
  out_hi <- get_pred_bounds(beta_aca, x0_realloc, vcov_aca, resdf_aca, bound = +1, alpha = 0.05)

  out_realloc <- c(out_pr, out_lo, out_hi)

  if (debug_mode) {
    print(out_realloc)
  }

  return(out_realloc)

})


# Define predictions for reallocation composition
delta_pred_aca <- reactive({

  init_ilrs <- ilr(init.comp())
  names(init_ilrs) <- paste0("ilr", 1:length(init_ilrs))

  realloc_ilrs <- ilr(Rcomp())
  names(realloc_ilrs) <- paste0("ilr", 1:length(realloc_ilrs))

  x0_delta <-
  make_x0(beta_aca, realloc_ilrs, input$sex, input$age, input$sep, input$puberty) -
  make_x0(beta_aca, init_ilrs, input$sex, input$age, input$sep, input$puberty)

  out_pr <- get_pred_bounds(beta_aca, x0_delta, vcov_aca, resdf_aca, bound =  0, alpha = 0.05)
  out_lo <- get_pred_bounds(beta_aca, x0_delta, vcov_aca, resdf_aca, bound = -1, alpha = 0.05)
  out_hi <- get_pred_bounds(beta_aca, x0_delta, vcov_aca, resdf_aca, bound = +1, alpha = 0.05)

  out_delta <- c(out_pr, out_lo, out_hi)

  if (debug_mode) {
    print(x0_delta)
    print(out_delta)
  }

  return(out_delta)

})

perc_change_aca <- reactive({ 100 * delta_pred_aca()[1] / init_pred_aca()[1] })



# ++++ predictions ++++

output$pred_plot_1 <- renderPlot({

  init_fat <- init_pred_fat()
  reall_fat <- reall_pred_fat()
  init_psy <- init_pred_psy()
  reall_psy <- reall_pred_psy()
  init_aca <- init_pred_aca()
  reall_aca <- reall_pred_aca()
  outc_labs <- c("Body fat (%)", "Psychosocial\n(scale score)", "Academic\n(NAPLAN score)")
  pred_type_labs <- c("Initial\n(before re-allocation)", "After\nre-allocation")


  plot_dat <-
    tibble(
      outc = rep(outc_labs, each = 2),
      pred_cat = rep("Predicitons", 6),
      pred_type = rep(pred_type_labs, 3)
    ) %>%
    bind_cols(
      .,
      as.data.frame(rbind(
        init_fat, reall_fat,
```

```
        init_psy, reall_psy,
        init_aca, reall_aca
      ))
    )

  plot_dat$outc <-
    factor(
      plot_dat$outc,
      levels = outc_labs
    )
  plot_dat$pred_type <-
    factor(
      plot_dat$pred_type,
      levels = pred_type_labs
    )

  if (debug_mode) {
    print(plot_dat)
  }

  plot_dat %>%
    ggplot(., aes(x = pred_type, y = V1)) + #, col = outc
    geom_point(size = 3) +
    geom_errorbar(aes(ymin = V2, ymax = V3), width = 0.06, linetype = 1, alpha = 0.75) +
    theme_bw() +
    facet_grid(outc ~ ., scales = "free") +
    labs(
      x = "Before or after time re-allocation",
      y = "Predicted value",
      col = "Health measure",
      title = "Predictions ",
      subtitle = "(initial and re-allocations)"
    ) +
    shiny_gg_theme()

})

output$pred_plot_2 <- renderPlot({

  delta_fat <- delta_pred_fat()
  delta_psy <- delta_pred_psy()
  delta_aca <- delta_pred_aca()
  outc_labs <- c("Body fat\n(%)", "Psychosocial\nhealth", "Academic\nperformance")

  plot_dat <-
    tibble(
      outc = outc_labs,
      pred_cat = rep("Predcited Difference", 3),
      pred_type = rep("Difference", 3)
    ) %>%
    bind_cols(
      .,
      as.data.frame(rbind(
        delta_fat, delta_psy, delta_aca
      ))
    )

  plot_dat$outc <-
    factor(
      plot_dat$outc,
      levels = outc_labs
    )

  if (debug_mode) {
    print(plot_dat)
  }

  ylo <- min(0, min(plot_dat$V2))
  yhi <- max(0, max(plot_dat$V3))
```

```
  plot_dat %>%
    ggplot(., aes(x = outc, y = V1)) + # , col = outc
    geom_hline(yintercept = 0, alpha = 0.25) +
    geom_point(size = 3) +
    geom_errorbar(aes(ymin = V2, ymax = V3), width = 0.06, linetype = 1, alpha = 0.75) +
    theme_bw() +
    ylim(ylo, yhi) +
    # facet_grid(outc ~ pred_cat, scales = "free") +
    labs(
      x = "Health measure",
      y = "Estimated difference",
      col = "Health measure",
      title = "Predicted difference ",
      subtitle = "(health measure at reallocation minus health measure at starting time-use)"
    ) +
    shiny_gg_theme()

})

observeEvent(input$sh_but, {

  if (debug_mode) {
    print(input$sh_but)
  }

  if (is.null(input$sh_but)) {
    shinyjs::hide(id = "plot1")
  } else if (input$sh_but %% 2 == 0) {
    shinyjs::hide(id = "plot1")
  } else {
    shinyjs::show(id = "plot1")
  }

})

output$ui1 <- renderUI({

  fat_val <- perc_change_fat()
  if (is.na(fat_val) | is.null(fat_val)) fat_val <- 0
  pm <- ifelse(fat_val > 0, "+", "")
  bx_col <- ifelse(fat_val > 0, "red", ifelse(fat_val < 0, "green", "black"))

  valueBox(
    value = paste0(pm, sprintf("%3.1f%%", fat_val)),
    subtitle = "Body fat % change",
    width = 4,
    color = bx_col
  )

})

output$ui2 <- renderUI({

  psy_val <- perc_change_psy()
  if (is.na(psy_val) | is.null(psy_val)) psy_val <- 0
  pm <- ifelse(psy_val > 0, "+", "")
  bx_col <- ifelse(psy_val > 0, "green", ifelse(psy_val < 0, "red", "black"))

  valueBox(
    value = paste0(pm, sprintf("%3.1f%%", psy_val)),
    subtitle = "Psychosocial change",
    width = 4,
    color = bx_col
  )

})

output$ui3 <- renderUI({
```

```
    aca_val <- perc_change_aca()
    if (is.na(aca_val) | is.null(aca_val)) aca_val <- 0
    pm <- ifelse(aca_val > 0, "+", "")
    bx_col <- ifelse(aca_val > 0, "green", ifelse(aca_val < 0, "red", "black"))

    valueBox(
      value = paste0(pm, sprintf("%3.1f%%", aca_val)),
      subtitle = "Academic change",
      width = 4,
      color = bx_col
    )

  })


  # ++++ close_app_action ++++

  # Close the R session when browser closes
  session$onSessionEnded(function() {
    stopApp()
  })


}  #server
```
