# Supplementary material for: Your best day: An interactive app to translate how time reallocations within a 24-hour day are associated with health measures
Source: PLoS One. 2022 Sep 7;17(9):e0272343. doi: 10.1371/journal.pone.0272343 (PMC9451088; doi:10.1371/journal.pone.0272343)
Supplement: S1 File — (PDF) [file pone.0272343.s004.pdf]

Glossary of Terms

Compositional data: Data consisting of two or more parts of a whole that convey relative information. Time-use data are compositional as they consist of activities (e.g., daily amounts of time spent in sleep, sedentary behavior and physical activity) which are mutually exclusive and exhaustive, summing to 24 hours every day.

Compositional data analysis: An analytical approach for compositional data first proposed in the 1980s by John Aitchison. Most commonly, compositional data analysis is performed by expressing the compositional data as log-ratio coordinates before their inclusion in standard statistical models.

Isometric log-ratio coordinates: Compositional data expressed as coordinates in Real space that include all relative information about the compositional parts and are suitable to be included in standard statistical models.

Compositional isotemporal substitution model: Regression model with log-ratio coordinates as explanatory variables and a health outcome as outcome variable that is used to estimate the difference in health outcome when time (e.g., 30 minutes) is reallocated between activities.

Compositional centre: The compositional equivalent of the average time-use composition. It is calculated by finding the geometric mean of each compositional part, and then normalising all the geometric means to a total amount (e.g. 100% or 24 hours).
