# Supplementary material for: Your best day: An interactive app to translate how time reallocations within a 24-hour day are associated with health measures
Source: PLoS One. 2022 Sep 7;17(9):e0272343. doi: 10.1371/journal.pone.0272343 (PMC9451088; doi:10.1371/journal.pone.0272343)
Supplement: Attachment — Submitted filename: Response to reviewers.docx [file pone.0272343.s005.docx]

Response to reviewers

Dear Editor,

As requested, we have addressed the reviewer's comments with special emphasis placed on:

1. Discussion of the health improvement mechanism drivers (please see response to R1.3)

2. Selection of the covariates used in the models (please see response to R2.2)

3. Differentiation between the association and  the causation (please see response to R2.4)

We have ensured the manuscript to meet PLOS ONE’s style requirements, as shown on the linked formatting sample documents.

In regard to data availability, unfortunately there are legal restrictions on public sharing of CheckPoint and LSAC data. To become a licensed data user for the CheckPoint and LSAC studies, researchers are required to sign a confidentiality deed, found here: https://ada.edu.au/wp-content/uploads/2019/11/Confidentiality-Deed-Poll-November-2019.pdf.

The confidentiality deed imposes a legal obligation to not share any unit-level data with anyone unless they also are registered data users. Researchers can apply to become registered data users by following the link provided below. https://growingupinaustralia.gov.au/data-and-documentation/accessing-lsac-data. Researchers are able to access these data in the same manner as the authors. The authors did not have any special access privileges.

As requested, the list of authors has been amended to meet style requirements.

| Reviewer comment | Response | Change to manuscript |
| --- | --- | --- |
| Reviewer 1  R1.1:This paper has important findings for user-defined time-use reallocations upon health outcomes via an app intervention. The paper finds that such reallocations lead to better objective and subjective health outcomes. In light of the fact that reallocations are user driven, app driven and continuous in nature, the results are novel and promising for users. | Thank you for the positive comment | No change |
| R1.2: On pages 5-6 of the manuscript, the point (empirical strategy) is made that log ratio form explanatory variables help to overcome perfect multicollinearity in the model (i.e., activity_1_time +…+ activity_t_time = 24 (hours)). It is then stated that log ratio form coefficients are more difficult to interpret within the model. Given this drawback, I suggest that the authors run an alternative model that uses the empirical strategy of leaving one category dummy variable out of the specification (i.e., create a reference group). In this manner, all coefficients are interpreted relative to the reference group. This would allow an alternative specification that maintains coefficient interpretability while also avoiding the dummy variable trap. | Thank you for this excellent suggestion – this was actually one of the methods we considered when preparing our analysis plan. We decided not to use this method because we wanted to have more flexibility in our modelling, i.e., we did not want to be limited to one-for-one (isotemporal) swaps between activities. We wanted the app user to have the choice for what kind of reallocations to make. Using our current method, the reallocations can be very complex, for example it is possible to increase time spent in an activity by 30 minutes while decreasing one activity by 10 minutes and decreasing another activity be 20 minutes. Our current method also overcomes the challenge of interpreting the log-ratio coefficients in a meaningful manner, thus we prefer to keep our current method. | No change |
| R1.3: I would like more discussion of what mechanism is driving the health improvements (e.g., based on past results from the literature). Is it the salience of seeing scheduled time use reallocations within an app that is driving behavioral change? Does the schedule create a commitment device that causes the behavioral change? Is it the act of planning and being deliberate in your time use decisions? | As suggested, we have added more discussion about potential mechanisms of successful behaviour change. | Page 19, para 2:  “The app may empower individuals and their supporting professionals/policy makers to make informed lifestyle choices according to what they value. It is designed to promote flexibility, autonomy and self-determination, which behaviour-change theory and empirical research show to be crucial to sustained lifestyle improvement (43, 44). By guiding the user to plan and be deliberate about their behaviour-change goals, the app may foster a sense of commitment and engagement.” |
| Reviewer 2 | | |
| R2.1 Dumuid et al. developed a web application so that users can easily estimate the association between time reallocations among various activities on body fat percentage, psychosocial health, and academic performance on a writing test in children, estimated using the compositional isotemporal substitution model. The manuscript is very well written and easy to follow the project goals and logic, and limitations are adequately discussed. The authors are careful in their use of language to not imply causal relationships from this method. The user-friendly open web application that produces estimates in interpretable outputs appears novel, and will make it easier to translate the compositional isotemporal substitution method to other outcomes and datasets.  In the dataset used to produce results in the application, a strength is that body fat percentage was measured (not self-report), and academic performance and psychosocial health assessed from standardized test/survey. 24h recalls were used to obtain self-report activity estimates from the children. The authors note this limitation in the discussion, which can be overcome with technological iterations and incorporated into the app in the future. I have downloaded the RShiny app and ran it locally to confirm that it works, and tested the web version as well. The directions were clear and it is easy to operate. | We were very happy to read that the reviewer successfully downloaded and tested the web version of our RShiny app. Thank you for the positive feedback. | No change. |
| R2.2: Covariates were self-reported (from child or parent). How did the authors decide which covariates to include in models? | We chose the covariates based on their known associations with time-use behaviour and health outcomes in children of this age. Text has been added to the methods to clarify this. | Page 9, para 2:  “Covariates were selected due to their known associations with children’s time-use behaviours and health outcomes. They included child’s sex, age, pubertal status, and family-level socioeconomic position.” |
| R2.3: The authors note in their discussion that the application can be helpful to public health promoters, medical practitioners, policy makers, etc. To push toward establishing causal relationships, this also seems useful to easily generate research hypotheses to test in interventions (at least the less complex allocations may be feasible to test). Given the limitations of traditional RCTs which would be impossible to assess all combinations of allocations, perhaps alternative designs could be used. There would be many challenges, but given a hypothesis generated by the application, perhaps in certain contexts, one may be able to use designs such as a micro-randomized trial to tease out the relative effectiveness of time reallocation scenarios in context of how time is already allocated. Reallocations could be randomized based on self-reported activities from mobile phones. Do the authors have thoughts on this use of their application? | This is an excellent idea. We would love to see the app implemented in future micro-RCTs. We have added a comment about this to the Implications for Practice section. | Page 20, para 1:  “The app could be used to generate research hypotheses about the effect of time reallocations which can be tested in randomised controlled trials. Large trials of digitally enabled cohorts (such as the GenV initiative now recruiting in Victoria, Australia (45) could provide opportunities for randomised trials to test efficacy of a further-refined app in achieving behaviour change and health gains.” |
| R2.4: The authors appropriately note the issue of causation in their limitations and that future iterations of the app should explicitly warn users to take caution in how they interpret results. I suggest that the authors add this now to the app, because assuming causation from assocational data is very common in the context of public health and policy, and given that they are among the targets of the application, this seems like an easy addition to help prevent misinterpretations going forward. Some notes on the self-reported nature of the activities seems warranted as well, or at least a prominent message and link to the paper once published to refer them to the methods. | Thank you for this good idea. We have added a note to the app interface to warn users of the current limitations (i.e., data are cross-sectional, therefore we cannot infer causation, time-use data were self-reported), and made note of this in the manuscript text.  We agree there are limitations to self-reported time use, but there are also advantages in terms of finding out what people are actually doing. Although perhaps more accurate, devices can only tell us about time spent in energy expenditure bands which has little relevance/meaning to everyday people. | Page 13, para 3:  “Our current app includes a brief disclaimer regarding the cross-sectional nature of the data, and that the activity data were derived from self-reports. Although the MARCA instrument has good-to-excellent measurement properties for children of this age…”  Added text to app interface:  “Differences in outcomes are not an indication of cause-and-effect as underlying data are cross-sectional. Also, note, time-use data were self-reported by study participants and may be subject to inaccuracies and biases.” |
| R2.5: The figure quality makes them difficult to read even after downloading the files in their original .tiff format. | We have uploaded new figures – the journal’s PACE figure tool requires the figure resolution to be reduced unfortunately. We hope readers will click on the web-link and use the app rather than relying on the figures. We are happy to supply higher resolution figures than allowed by the PACE tool to the journal editors upon request. | New figures uploaded, following correction by PLOS’ online PACE tool. |

New references added during the revision process:

43.	Yeager DS, Dahl RE and Dweck CS. Why interventions to influence adolescent behavior often fail but could succeed. Perspect Pych 2018;13:101-122.

44.	Gillison FB, Rouse P, Standage M, Sebire SJ, Ryan RM. A meta-analysis of techniques to promote motivation for health behaviour change from a self-determination theory perspective. Health Psych Rev. 2019;13(1):110-30.

45.	Wake M, Hu YJ, Warren H, Danchin M, Fahey M, Orsini F, Pacilli M, Perrett KP, Saffery R, Davidson A. Integrating trials into a whole-population cohort of children and parents: statement of intent (trials) for the Generation Victoria (GenV) cohort. BMC Med Res Methodol. 2020;20(1):238
